# The Marriage of Syphilitics

**Published:** 1919-07-19

**Authors:** C. O. Hawthorne


### THE EDITOR'S LETTER-BOX.

**[Correspondence on all subjects is invited, but we cannot in any way be responsible for the opinions expressed by our correspondents, who must give their name and address as a guarantee of good faith, but not necessarily for publication. Correspondents are reminded that brevity of style and conciseness of statement greatly facilitate early insertion.]**

### THE MARRIAGE OF SYPHILITICS.

*To the Editor of* The Hospital.

Sir,—In his letter of the 12th inst. (p. 395) your contributor defines the event which demands, in his judgment, a breach of professional confidence with a view to prevent the marriage of a person "suffering from venereal disease." The intervention is to take place when, and only when, the disease is "certain to be communicated" to the other party to the contract. Add to this limitation the necessity (1) that the sufferer, before his marriage, shall consult a doctor, (2) that he shall inform the doctor of his intended marriage, and (3) that he shall announce also the name and address of his prospective spouse, and your readers will judge for themselves the frequency with which the scheme would have an opportunity of coming into effective operation. It is for the exceptional occasion established by the above conditions that your contributor asks us to pay a "high price" and to sacrifice the "honourable tradition" of the profession, while, at the same time, we are to do nothing to prevent the marriage of an epileptic, insane, or tuberculous person, nor, indeed, of a syphilitic person when the disease is not "certain to be communicated." My submission, on the other hand, has been that the medical profession is not bound, either legally or ethically, to reveal voluntarily the confidences of the consulting-room, and that its true function in relation to marriage is to educate public opinion to the conviction that the prevention of the spread of disease by marriage is to be secured by the presentation, by each party to the contract, of a health certificate, having the value of that expected in the case of a life-insurance policy. This is the broad issue of the present controversy, and I am content to leave the choice to the popular judgment.—Yours faithfully,

C. O. Hawthorne.

